# Resolution of Persistent Fever and Systemic Inflammation in a Metastatic Gastrointestinal Stromal Tumour (GIST) With Imatinib

**DOI:** 10.7759/cureus.97356

**Published:** 2025-11-20

**Authors:** Sumaya Akter, K S M Zibran Zalis Gaznavee, Rukhshana Rabbani, Shadman Sakib Rahman, Stergios Boussios

**Affiliations:** 1 Internal Medicine, Medway NHS Foundation Trust, Gillingham, GBR; 2 Critical Care Medicine, Medway NHS Foundation Trust, Gillingham, GBR; 3 Clinical Oncology, Royal Marsden Hospital, Sutton, GBR; 4 Oncology, Medway NHS Foundation Trust, Gillingham, GBR

**Keywords:** gastrointestinal stromal tumour (gist), imatinib therapy, metastatic disease, paraneoplastic fever, systemic inflammation, tyrosine kinase inhibitor (tki)

## Abstract

This case highlights the critical role of imatinib in managing gastrointestinal stromal tumours (GISTs), especially in metastatic cases where surgical resection is not feasible. In addition to controlling tumour growth, imatinib appears to help manage paraneoplastic symptoms such as systemic inflammation. The patient’s atypical presentation with persistent fever and elevated inflammatory markers, which resolved with imatinib, underscores the need for clinicians to consider its immune-modulating properties in the management of GIST.

A comparison with traditional treatments, such as chemotherapy and surgical resection, demonstrates the superior efficacy of imatinib in metastatic GISTs. While surgery remains essential for localized disease, imatinib has transformed the treatment landscape for metastatic and unresectable GIST, significantly enhancing patient outcomes.

Further prospective clinical studies are warranted to deepen our understanding of the immune-modulating effects of imatinib and to optimize its use in GIST patients presenting with inflammatory symptoms.

## Introduction

Gastrointestinal stromal tumours (GISTs) are rare mesenchymal tumours, accounting for approximately 3-5% of all soft tissue sarcomas and 1-3% of all gastrointestinal tumours [1,2]. GISTs are most commonly located in the stomach (50-70%) and small intestine (20-30%) [3], but they can also arise in extra-gastrointestinal sites such as the mesentery and omentum. These tumours are primarily driven (around 80%) by mutations in the c-KIT or PDGFRA genes, leading to the activation of tyrosine kinase pathways that promote tumour growth and survival [4]. The identification of these mutations has facilitated the development of targeted therapies, particularly imatinib, a tyrosine kinase inhibitor (TKI), for the treatment of metastatic or unresectable GISTs [5].

A small percentage of patients may have systemic inflammatory manifestations, such as fever, exhaustion, and elevated inflammatory markers, even though GISTs usually manifest as gastrointestinal symptoms like abdominal pain, bleeding, or obstruction [6-8]. Diagnostic uncertainty may result from mistaking these for infections or toxicity from treatments. It is believed that cytokine-mediated processes, specifically the overproduction of interleukin-6 (IL-6) and other pro-inflammatory mediators released by tumour or stromal cells, are responsible for the paraneoplastic inflammatory syndromes linked to GIST [7,8]. Despite being uncommon, these presentations have clinical significance since they can mask the underlying diagnosis and affect treatment choices.

This report details the case of a patient with metastatic GIST who experienced systemic inflammation and a persistent fever after temporarily stopping imatinib therapy because of treatment-related fatigue. Imatinib’s antitumor efficacy and possible anti-inflammatory effects were highlighted by the quick resolution of symptoms upon reintroduction. Therefore, this case emphasises the crucial role of Imatinib to address the paraneoplastic symptoms like cytokine-driven inflammation in GIST.

## Case presentation

**Figure 1:**
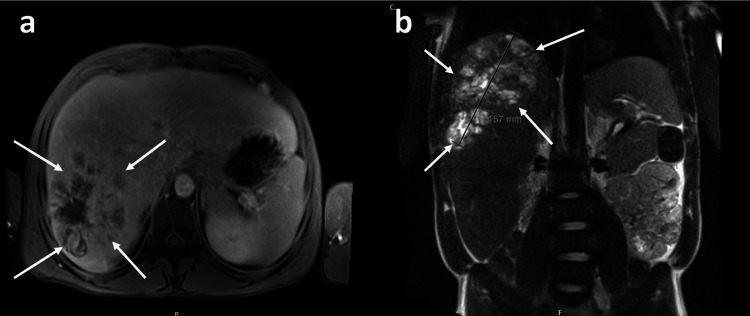
MRI of the liver (a) Axial view: There’s a distinct dark lesion in the liver, which stands out from the surrounding tissue. It is irregular in shape and appears hypointense compared to the normal liver parenchyma and corresponds to a metastatic deposit. (b) Coronal view: The large mass in the liver region is clearly marked with a measurement of 157 mm, suggesting a significant abnormality. Its size and location are consistent with a metastatic GIST.

A man in his 30s was recently diagnosed with metastatic epithelioid GIST (Figure 1), where the primary site was the lesser curvature of the stomach. The metastatic disease had spread to his liver, rendering the tumour inoperable due to extensive metastasis. He began treatment with imatinib, starting his first cycle the month following his diagnosis.

After 23 days, he presented to the hospital with persistent fever, shaking, and fatigue that had developed over several days. Upon admission, he was febrile, with a high temperature of 39 °C. Laboratory tests revealed significantly elevated inflammatory markers, raising suspicion of a potential systemic infection.

Investigations

The patient’s investigations revealed significant abnormalities in inflammatory and haematological markers (Table 1). A C-reactive protein (CRP) level of 501.2 mg/L indicated a profound inflammatory response. Haematological analysis showed a markedly elevated white blood cell (WBC) count of 56.4 × 10^9/L, predominantly neutrophils (54.0 × 10^9/L), suggesting a systemic reaction likely attributable to infection, inflammation, or malignancy-associated processes. The platelet count was elevated at 454 × 10^9/L, consistent with reactive thrombocytosis, a condition commonly associated with systemic inflammation. Haemoglobin was markedly low at 67 g/L, indicating iron deficiency anaemia. This anaemia was likely multifactorial, potentially due to chronic inflammation, the underlying disease, chemotherapy effects, or bone marrow suppression.

**Table 1:** Laboratory results MCV: mean corpuscular volume; GFR: glomerular filtration rate; CRP: C-reactive protein; ALP: alkaline phosphate; ALT: alanine transaminase

Test	Result	Unit	Reference value
Reticulocyte Count	58	10^9/L	25-85
WBC	56.4	10^9/L	4.0-11.0
RBC	2.48	10^12/L	4.50-5.50
Haemoglobin	67	g/L	130-170
Haematocrit	0.22	L/L	0.40-0.50
MCV	89.8	fL	80.0-100.0
Platelet Count	454	10^9/L	150-410
Automated Neutrophil Count	54.0	10^9/L	2.0-7.0
Automated Lymphocyte Count	1.4	10^9/L	1.0-4.0
Estimated GFR	72	mL/min/1.73m^2	>90
Creatinine	103	umol/L	59-104
Sodium	132	mmol/L	133-146
Potassium	4.7	mmol/L	3.5-5.3
High-Sensitivity CRP	501.2	mg/L	0.0-5.0
Albumin	20	g/L	35-50
Total Bilirubin	19	umol/L	0-21
ALP	404	U/L	30-130
ALT	21	U/L	<50
Urea	14.9	mmol/L	2.5-7.8

**Figure 2:**
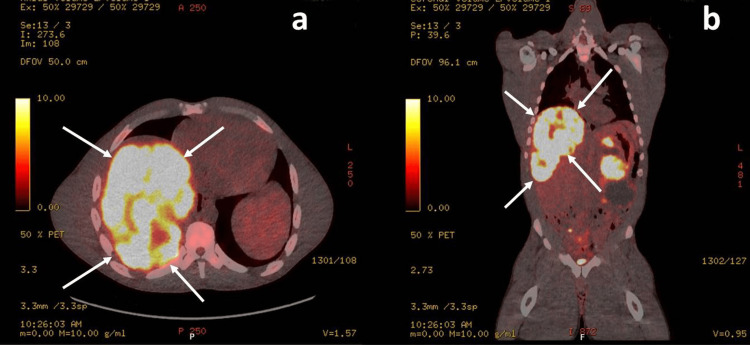
PET scan of the liver (a) Axial view: It shows a focal hotspot in the liver, consistent with the lesion’s location in the MRI. (b) Coronal view: There is a prominent area of high metabolic activity in the liver region, shown as bright yellow/white. This corresponds to the metastatic GIST, which aligns with the large mass seen in the MRI. PET: positron emission tomography; GIST: gastrointestinal stromal tumour

Further diagnostic tests were conducted to investigate the cause of the systemic inflammatory response. Blood cultures, as well as comprehensive fungal and viral screenings, did not identify any pathogenic organisms (Table 2), effectively ruling out bacterial, fungal, or viral infections as the source of inflammation. Imaging studies, including a CT scan of the chest, abdomen, and pelvis, showed no evidence of abscesses, mass effects, or other infectious foci. A chest X-ray was clear, and a liver magnetic resonance imaging (MRI) revealed partial regression of a metastatic lesion, shrinking from 23 cm to 16 cm, with no evidence of liver abscess formation (Figure 2).

**Table 2:** Blood culture, viral, and fungal screening results RSV: respiratory syncytial virus

Parameter	Result
Blood culture	No growth after 5 days of incubation
Flu A	NOT detected
Flu B	NOT detected
RSV	NOT detected
Aspergillus antigen test	Negative
Beta Glucan test	Negative

Microscopic examination of a blood film provided additional insights, showing neutrophilia with toxic granulations, a left shift, and occasional nucleated red blood cells. No blasts were observed, effectively ruling out leukemic processes. These findings suggested that the observed blood abnormalities were reactive, likely related to the underlying disease process or recent chemotherapy.

Collectively, the investigations demonstrated a profound inflammatory state without a clearly identifiable infectious aetiology, prompting further exploration of non-infectious causes for the systemic response.

Treatment

The patient initially received antipyretics and intravenous piperacillin-tazobactam for bacterial infection coverage, which was later escalated to meropenem and caspofungin to address the possibility of fungal infections due to persistent symptoms. When fever spikes continued, vancomycin and ciprofloxacin were added based on recommendations from the microbiology team. Persistent anaemia was managed with multiple red blood cell transfusions. Despite these interventions, the fever persisted. Following a multidisciplinary discussion, antibiotics were discontinued, and treatment with imatinib was resumed.

Outcome and follow-up

**Figure 3:**
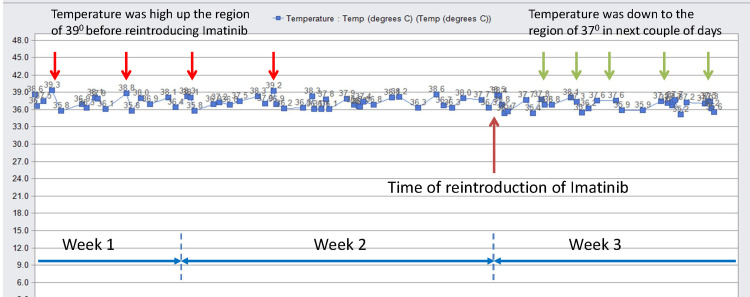
Temperature chart from the electronic patient record before and after reintroducing imatinib

Following the resumption of imatinib treatment, the patient’s fever subsided, and he demonstrated significant clinical improvement. By the time of discharge, he was stable, with no further fever spikes (Figure 3), fatigue, or drops in haemoglobin, and had regained his energy level. He is scheduled for twice-weekly blood tests at the Cancer Day Unit (GDU) and will be closely monitored by his treating oncologist.

## Discussion

Surgical resection as a primary treatment strategy

Historically, surgical resection was the standard treatment for GISTs, particularly in localized cases [9]. Surgery offers the potential for a cure when the tumour is resectable and confined to a specific area. However, for patients with metastatic disease or tumours deemed inoperable due to their size, location, or involvement of vital structures, surgery is often not a viable option. In such cases, the prognosis was historically poor until the advent of imatinib [10]. The case discussed here exemplifies this scenario, as liver metastasis and the size of the lesions rendered surgical resection impossible. Consequently, imatinib therapy was initiated as a palliative treatment, given its proven efficacy in controlling disease progression [11].

Systemic therapy evolution: from chemotherapy to tyrosine kinase inhibitor (TKIs)

Before the introduction of imatinib, chemotherapy was the primary treatment for metastatic GIST, but it yielded poor response rates. Agents like doxorubicin and streptozotocin demonstrated limited efficacy and were often associated with significant toxicity. The identification of c-KIT and PDGFRA mutations in GISTs led to the development of targeted therapies such as imatinib [12]. Imatinib, an oral TKI, selectively inhibits the c-KIT and PDGFRA receptors, which play key roles in the pathogenesis of GISTs. By targeting these pathways, imatinib effectively reduces tumour size, improves progression-free survival (PFS), and alleviates symptoms in patients with metastatic GIST [13,14]. In this case, partial regression of liver metastases was observed after resuming Imatinib therapy, underscoring its efficacy in controlling tumour progression.

Imatinib's role in managing systemic inflammation

A critical aspect of this case is the patient’s presentation with fever, fatigue, and elevated inflammatory markers - symptoms that are uncommon in typical GIST cases [10]. These symptoms could be attributed to a paraneoplastic syndrome, often linked to the release of cytokines or bioactive molecules by tumours. In GISTs, cytokines such as interleukin-6 (IL-6) and tumour necrosis factor-alpha (TNF-α), are believed to contribute to systemic inflammation [15].

Imatinib not only inhibits tumour growth but has also been reported to reduce the production of inflammatory cytokines and modulate immune responses [11]. From a pathophysiological perspective, this dual action is highly significant. By targeting the BCR-ABL tyrosine kinase and other kinases, such as c-KIT and PDGFR, imatinib directly disrupts proliferative signalling pathways critical for tumour cell survival and growth. Simultaneously, its ability to reduce inflammatory cytokine production mitigates the chronic inflammatory microenvironment that often promotes tumour progression and immune evasion.

Furthermore, imatinib’s immunomodulatory effects may involve the restoration of T-cell activity and suppression of regulatory T cells (Tregs), which tumours frequently exploit to dampen anti-tumour immunity. These combined mechanisms suggest that Imatinib functions not only as a direct antineoplastic agent but also as a modulator of the tumour microenvironment, enhancing the host’s immune surveillance and response.

In this case, the patient’s systemic symptoms, including fever, resolved after the re-initiation of Imatinib therapy. This indicates that imatinib played a role not only in tumour control but also in modulating the paraneoplastic inflammatory syndrome. This stands in contrast to traditional treatments, such as antibiotics or corticosteroids, which do not address the underlying cause of inflammation.

Mechanisms of imatinib and its impact on tumour biology and inflammation

Imatinib’s efficacy in GISTs stems from its ability to inhibit two key receptor tyrosine kinases, c-KIT and PDGFRA, both of which are frequently mutated in these tumours [16]. Mutations in c-KIT, found in approximately 70-85% of GISTs, result in continuous activation of downstream signalling pathways such as mitogen-activated protein kinases (MAPK), phosphatidylinositol 3-kinase (PI3K)/protein kinase B (Akt), and Janus kinase/signal transducer and activator of transcription (JAK/STAT). These pathways drive uncontrolled cell proliferation, survival, and resistance to apoptosis. Imatinib binds to the adenosine triphosphate (ATP)-binding pocket of these mutated tyrosine kinases, preventing their activation and halting the propagation of oncogenic signals.

By inhibiting c-KIT, imatinib induces apoptosis in tumour cells, effectively reducing tumour burden and limiting disease progression. Similarly, its inhibition of PDGFRA - mutated in 5-10% of GISTs - provides an alternative therapeutic target, particularly in cases where c-KIT mutations are absent [17]. This dual mechanism of action makes imatinib highly effective, particularly as a first-line treatment for unresectable or metastatic GISTs [16]. Clinical studies have demonstrated substantial improvements in PFS and overall survival with imatinib therapy.

Beyond its direct effects on tumour cells, imatinib may modulate the tumour microenvironment by reducing stromal support and angiogenesis, both of which are critical for tumour progression. However, resistance to Imatinib often develops after two to three years of therapy, primarily due to secondary mutations in c-KIT or PDGFRA that restore kinase activity [5,17]. This underscores the need for continued research into second- and third-line therapies to address resistance.

Imatinib has been shown to inhibit the production of pro-inflammatory cytokines, including TNF-α, IL-12(p40), and IL-1α, by synovial fluid mononuclear cells, demonstrating its capacity to modulate inflammation through cytokine suppression, even beyond oncologic settings [18]. In GIST, imatinib has been reported to reduce intratumoral expression of the immunosuppressive enzyme IDO and to alter T-cell profiles by increasing the CD8⁺/Treg ratio, thereby reshaping the immune milieu. Although cytokine levels, such as IL-1β, IL-6, TNF-α, IL-17, and IL-10, were not significantly changed in that particular study, these findings provide strong mechanistic support for the immunomodulatory role of imatinib [19]. Furthermore, increased expression of stem cell growth factor α (SCGFα) in the stromal component of GISTs following imatinib therapy suggests an effect on monocyte and macrophage recruitment, indicating that imatinib impacts not only tumour cells directly but also the inflammatory and stromal compartments of the tumour microenvironment [20].

## Conclusions

Overall, imatinib has revolutionized the management of GISTs, serving as a model for targeted cancer therapies that exploit specific molecular vulnerabilities. In addition to its anti-tumour effects, imatinib possesses immune-modulatory properties that can reduce the systemic inflammation associated with GISTs. In the present case, the resolution of fever and normalization of inflammatory markers following the resumption of imatinib therapy suggest that the drug exerts a dual effect, targeting both tumour growth and inflammation.

However, it is necessary to acknowledge that a single case report (especially as a similar case has a low percentage) might not explicitly establish a definitive imatinib’s antitumor and anti-inflammatory effects in the given context. For further insight into inflammatory manifestations in GIST and their response to tyrosine kinase inhibitors, more documentation and research are needed.
